# Genetic diversity and population structure of *Plasmodium vivax* in Central China

**DOI:** 10.1186/1475-2875-13-262

**Published:** 2014-07-09

**Authors:** Yaobao Liu, Sarah Auburn, Jun Cao, Hidayat Trimarsanto, Huayun Zhou, Karen-Ann Gray, Taane G Clark, Ric N Price, Qin Cheng, Rui Huang, Qi Gao

**Affiliations:** 1 Medical College of Soochow University, Suzhou, Jiangsu, People’s Republic of China; 2 Jiangsu Institute of Parasitic Diseases, Key Laboratory of Parasitic Disease Control and Prevention (Ministry of Health), Jiangsu Provincial Key Laboratory of Parasite Molecular Biology, Wuxi, Jiangsu, People’s Republic of China; 3 Global and Tropical Health Division, Menzies School of Health Research and Charles Darwin University, Darwin, Northern Territory, Australia; 4 Eijkman Institute for Molecular Biology, Jakarta, Indonesia; 5 Drug Resistance and Diagnostics, Australian Army Malaria Institute, Weary Dunlop Drive, Gallipoli Barracks, Enoggera, Queensland, Australia; 6 Department of Pathogen Molecular Biology, London School of Hygiene and Tropical Medicine, London WC1E 7HT, UK; 7 Centre for Tropical Medicine, Nuffield Department of Clinical Medicine, University of Oxford, Oxford, UK

**Keywords:** *Plasmodium vivax*, China, Anhui, Jiangsu, Population genetics, Population structure, Transmission, Diversity

## Abstract

**Background:**

In Central China the declining incidence of *Plasmodium vivax* has been interrupted by epidemic expansions and imported cases. The impact of these changes on the local parasite population, and concurrent risks of future resurgence, was assessed.

**Methods:**

*Plasmodium vivax* isolates collected from Anhui and Jiangsu provinces, Central China between 2007 and 2010 were genotyped using capillary electrophoresis at seven polymorphic short tandem repeat markers. Spatial and temporal analyses of within-host and population diversity, population structure, and relatedness were conducted on these isolates.

**Results:**

Polyclonal infections were infrequent in the 94 isolates from Anhui (4%) and 25 from Jiangsu (12%), with a trend for increasing frequency from 2008 to 2010 (2 to 19%) when combined. Population diversity was high in both provinces and across the years tested (*H*_E_ = 0.8 – 0.85). Differentiation between Anhui and Jiangsu was modest (*F’*_*ST*_ = 0.1). Several clusters of isolates with identical multi-locus haplotypes were observed across both Anhui and Jiangsu. Linkage disequilibrium was strong in both populations and in each year tested (*I*_A_^S^ = 0.2 – 0.4), but declined two- to four-fold when identical haplotypes were accounted for, indicative of occasional epidemic transmission dynamics. None of five imported isolates shared identical haplotypes to any of the central Chinese isolates.

**Conclusions:**

The population genetic structure of *P. vivax* in Central China highlights unstable transmission, with limited barriers to gene flow between the central provinces. Despite low endemicity, population diversity remained high, but the reservoirs sustaining this diversity remain unclear. The challenge of imported cases and risks of resurgence emphasize the need for continued surveillance to detect early warning signals. Although parasite genotyping has potential to inform the management of outbreaks, further studies are required to identify suitable marker panels for resolving local from imported *P. vivax* isolates.

## Background

China has made considerable progress in reducing the burden of malaria, since the launch of the National Malaria Control Programme in 1955. Despite two major outbreaks in the 1960s and 1970s, the incidence of malaria cases has dropped steadily from an estimated 6.79 million cases in 1954 to less than 15,000 in 2009 [1]. In July 2010, the National Malaria Elimination Programme was established, with the goals to achieve elimination in all regions, except the Myanmar border region of Yunnan Province, by 2015 and nation-wide by 2020 [2].

Across China, marked heterogeneity is observed in malaria incidence, and in the distribution of *Plasmodium vivax* and *Plasmodium falciparum* cases. Historically, *P. falciparum* was endemic in the south, whilst *P. vivax* was more prevalent in the temperate central regions. The implementation of malaria control interventions has completely interrupted transmission of *P. falciparum* in Central China in the past decade. However, owing in part to the parasite’s complex transmission dynamics, ability to relapse weeks or months after initial infection, and challenges in diagnosing low parasitaemia infections, *P. vivax* has proven to be more difficult to eliminate than *P. falciparum*[3]. With rising levels of drug resistance, and accumulating reports of severe, life-threatening disease, *P. vivax* continues to present a major public health threat [4-7]. In the early 2000s, outbreaks of *P. vivax* infection in the Central China provinces of Anhui, Jiangsu and Henan highlighted the risk of resurgence, and the importance of maintaining a strong surveillance system [8]. The regions along the Huai River were most affected by the resurgence, particularly in Anhui Province (2000-2006), which accounted for more than half of the total annual malaria cases in China in those years [9]. Although the number of *P. vivax* cases in Central China has declined steadily since 2006, the threat of future resurgence remains.

A recent *P. vivax* genotyping study in Sabah, Malaysia, demonstrated that focal epidemic expansions may become more frequent in areas with unstable transmission, characteristics of pre-elimination settings [10]. The risk of resurgence in these unstable transmission settings is a major threat to elimination. Imported malaria is of particular concern in this context as it may be an important contributor to local outbreaks. In Central China, importation of *P. vivax* cases across provincial boundaries or internationally are significant contributors to the overall *P. vivax* incidence in provinces such as Jiangsu [11-13]. Relative to the temperate strains endemic to Central China, the higher relapse rate of the isolates imported from tropical regions in the south and internationally may influence local transmission dynamics [12,14]. Information on the diversity and transmission dynamics of the *P. vivax* population in Central China can potentially provide insights into the changing dynamics of declining malaria incidence and the progression of elimination, as well as the effect of imported cases on the likelihood of successful elimination in this region.

To obtain the baseline molecular epidemiology status and assess the risks of further *P. vivax* resurgence in Central China, seven polymorphic short tandem repeat (STR) markers were genotyped to determine the local patterns of diversity and transmission in isolates collected from Anhui and Jiangsu Province between 2007 and 2010. In addition, provisional assessment of the utility of the STR marker panel to distinguish local from imported *P. vivax* cases was undertaken by comparison of the genetic profiles of central Chinese isolates with those from a selection of isolates imported from Southern China and a range of international sites.

## Methods

### Ethics

All samples were collected with written informed consent from the patient, parent or legal guardian (individuals < 18 years of age). The study was approved by the Institutional Review Board of Jiangsu Institute of Parasitic Diseases (IRB00004221), Wuxi, China.

### Study sites and sample collection

**Figure 1:**
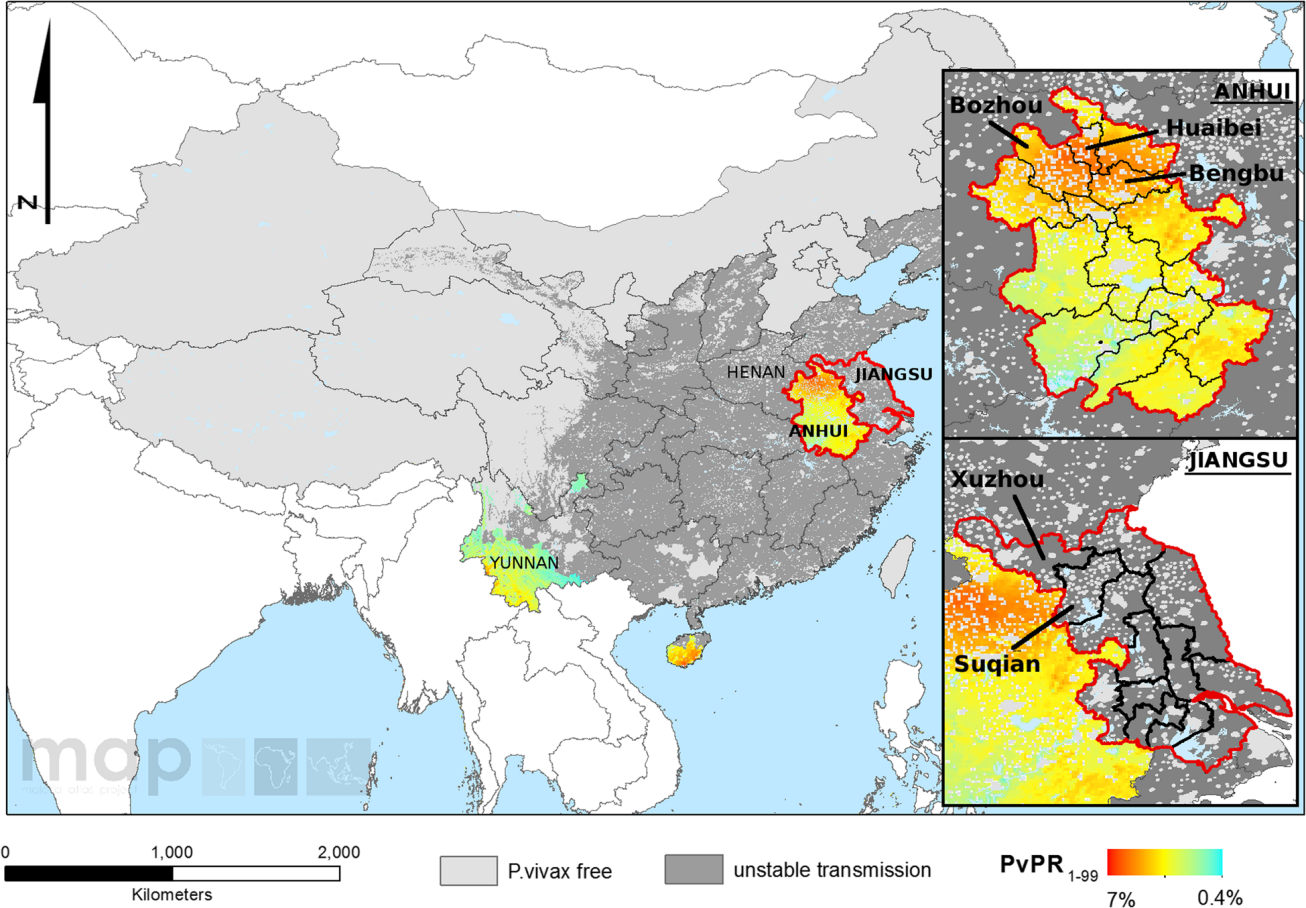
**Spatial distribution of *P. vivax *endemicity in 2010 in China.** This map was generated by Zhi Huang, Malaria Atlas Project, University of Oxford. The colour scale reflects the age-standardized *P. vivax* parasite rate (PvPR), which describes the estimated proportion of the general population that are infected with *P. vivax* at any one time, averaged over the 12 months of 2010 within the spatial limits of stable transmission [17]. The main figure presents the distribution across all China, with labelling of the provinces from which samples are represented in this study. The plots in the right-hand boxes indicate the prefectures from which the majority of malaria cases in Anhui Province (top) and Jiangsu Province (bottom) originated.

A summary of the study sites, sample sizes, dates of collection, and patient characteristics are presented in Table 1. The focal study sites were in Anhui and Jiangsu Province, located in Central China (Figure 1). Sampling was undertaken by passive case detection and a *P. vivax* transmission study in Central China.

**Table 1:** Sample details

**Country**	**Province**	**Year**	**Malaria incidence**^**1**^	**No. samples (n**^**2**^**)**	**Median age, years (Range)**	**% Males**
**China**	**Anhui**	2007	5.00 [9]	5 (5)	32 (20-46)	0 %
		2008	2.42 [15]	47 (45)	36 (10-70)	51 %
		2009	1.19 [15]	31 (28)	33 (6-69)	65 %
		2010	0.28 [16]	24 (16)	27 (6-69)	67 %
		**2007-10**	**-**	**107 (94)**	**34 (6-70)**	**56 %**
	**Jiangsu**	2008	0.09 [15]	17 (9)	29 (5-72)	53 %
		2009	0.05 [15]	18 (11)	53 (17-73)	50 %
		2010	0.05 [13]	8 (5)	34 (20-62)	75 %
		**2008-10**	**-**	**43 (25)**	**40 (5-73)**	**56 %**
	**Henan**	2010	-	2 (2)	(12, 25)	50 %
	**Yunnan**	2010	-	2 (2)	(26, 55)	100 %
**India**		2010	-	1 (1)	(22)	0 %
**Nigeria**		2010	-	2 (1)	(21, 45)	100 %
**Pakistan**		2010	-	2 (2)	(24, 45)	100 %
**PNG**		2010	-	1 (1)	(35)	100 %
**South Africa**		2010	-	1 (0)	(48)	100 %
**All**		**2007-10**	-	**161 (128)**	**-**	**-**

^1^Annual confirmed cases per **10,000** population (reference in square brackets). n^**2**^ = number of samples with ≥ 5 markers successfully genotyped.

**Figure 2:**
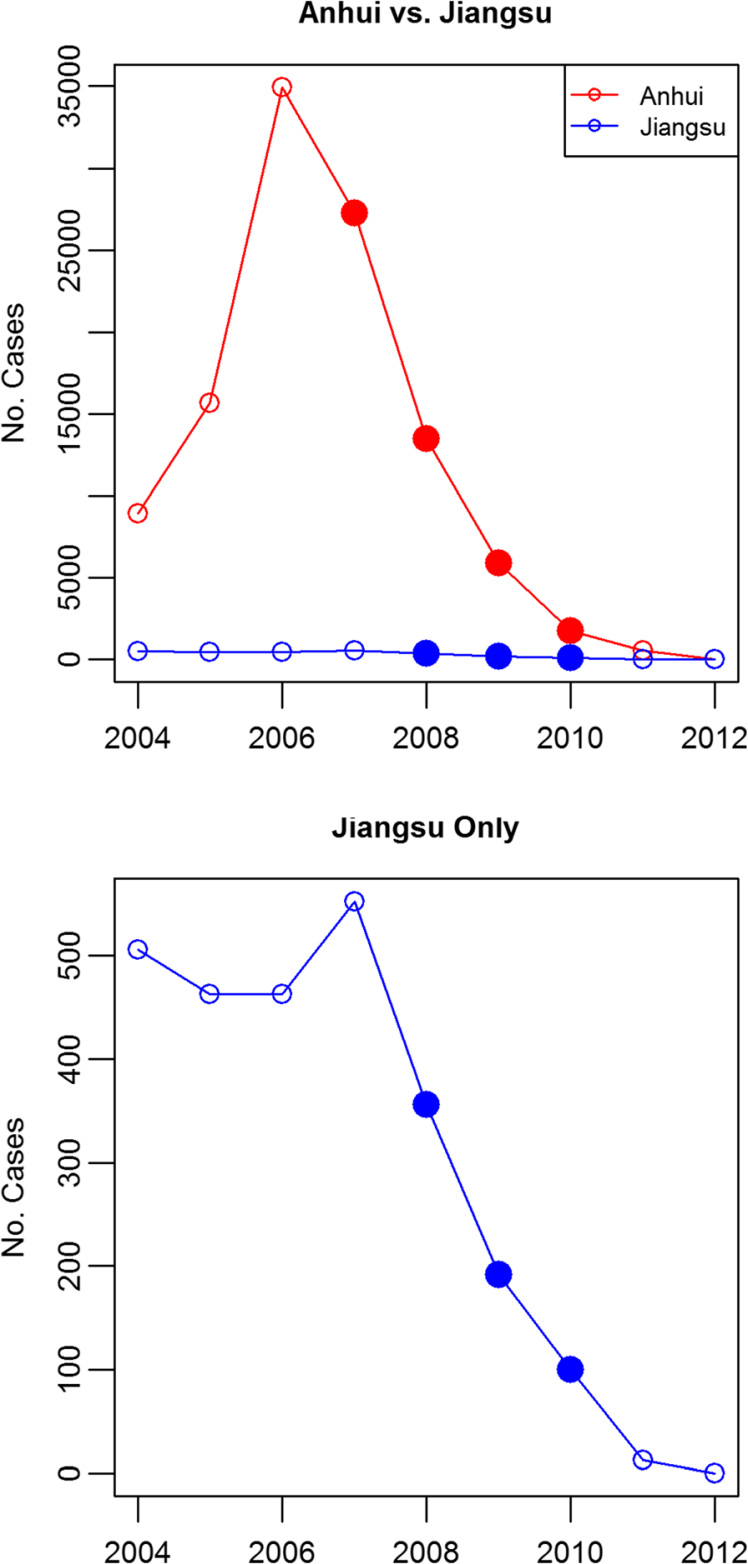
**Incidence of indigenous *P. vivax *cases in Anhui and Jiangsu Province.** Data source: Annual case report of parasitic diseases from Ministry of Health and Health Department of Jiangsu Province. Sampling time points are indicated by filled in circles.

Anhui province covers an area of 139,600 km^2^ divided into 17 prefecture-level divisions and 105 counties. In 2010, the population was estimated at 59.5 million people [18], living in the fertile agricultural regions along the Huai River. In the early 2000s, an outbreak led to an increase in *P. vivax* cases, peaking in 2006, with 34,984 malaria (all species) cases [19], before it was suppressed with subsequent steady decline in reported cases (Figure 2). As a result of the outbreak, Anhui experienced the highest incidence of malaria (all species) in the country between 2005 and 2009 [15]. Blood samples were collected from *P. vivax* patients in Anhui between 2007 to 2010, during which the incidence of malaria fell from approximately 5.0 to 0.28 per 10,000 population [9,15,20]. The majority of patients came from Bengbu, Bozhou and Huaibei prefectures, located in the north of the Province (Figure 1). In these regions, the climate is temperate, and malaria transmission is seasonal, peaking in July and August.

Jiangsu province covers an area of 102,600 km^2^ divided into 13 prefecture-level divisions and 106 counties. In 2010, the population was estimated at 78.7 million people [18]. In contrast to Anhui, Jiangsu is a major hot spot for economic development, with major industries in electronics, chemicals and textiles. Although overall malaria incidence was lower than in Anhui, Jiangsu was also affected by the *P. vivax* resurgence in the early 2000s (Figure 2). As with Anhui, blood samples were collected from *P. vivax* patients in Jiangsu between 2008 and 2010, during which malaria incidence ranged from approximately 0.09 to 0.05 per 10,000 population [20,21]. The majority of patients came from Xuzhou and Suqian prefectures, located in the north of the Province (Figure 1). These regions experience a temperate climate, with seasonal malaria transmission peaking in mid-July to early August [22].

Samples from Anhui and Jiangsu Province were collected from patients confirmed as locally acquired by epidemiologic case investigation. Additional samples for provisional assessment of the markers’ ability to differentiate between local and cross-province or internationally imported cases were collected from patients visiting or returning from Henan and Yunnan Province in central and south China, respectively, and from other countries. Approximately 100 μL capillary blood samples were collected on filter paper from patients attending local hospitals who were microscopy positive for *P. vivax*. Details on recent travel history and home address were recorded for each patient in order to determine the most likely site where the infection was acquired.

### DNA extraction and PCR-based species identification

Extraction of genomic DNA from blood on filter papers was conducted using QIAGEN QIAamp DNA Mini Kits and a QIAcube robot (QIAGEN, Crawley, U.K.). The manufacturer’s protocol (QIAamp DNA Mini and Blood Mini Handbook 2E) was followed, except only one 5 mm circle was extracted per sample, and eluted to a 100 μL volume. Detection for Plasmodium species was undertaken using the method described previously [23]. DNA samples that were determined positive for *P. vivax* were used for genotyping.

### Genotyping

Genotyping was undertaken at seven previously described short tandem repeat markers including Pv3.27, msp1F3, MS1, MS5, MS8, MS10 and MS16 [24,25]. These markers are included in a consensus panel selected by Country Partners within the Asia Pacific Malaria Elimination Network (APMEN) Vivax Working Group. The APMEN Vivax Working Group is a body of 14 country partners that have acknowledged the utility of genotyping to inform on the parasite’s transmission dynamics and patterns of spread within and across borders, developing a consensus with which to compare and contrast parasite populations from across the Asia-Pacific region.

The Pv3.27, MS16 and msp1F3 loci were amplified using the methods described by Gray *et al.*[26] entailing two rounds of amplification, with a multiplex first round reaction and separate second round reactions. The MS1, MS5, MS8 and MS10 loci were amplified using a single round of PCR following the protocol described by Gunawardena *et al.*[27]. The primer sequences for each marker are provided in Additional file 1.

The final labelled PCR products were diluted and sized by denaturing capillary electrophoresis on an ABI 3100 Genetic Analyzer with GeneScan LIZ-500 (Applied Biosystems) internal size standards. Capillary electrophoresis was undertaken at the QIMR Berghofer Medical Research Institute Scientific Services Analytic Facility. Fragment size was determined using Genescan and Peak Scanner software™ Version 1.0 (Applied Biosystems). All electropherogram traces were additionally inspected manually. Negative controls (no DNA and DNA from non-parasitized human blood) and positive controls (DNA from standard strains PVQ, AMRU1 and AMRU2) were used in every run. For each isolate, at each locus, the predominant allele (highest intensity peak), and any additional alleles with peak height at least one-third of the height of the predominant allele were scored [28]. Genotyping success was defined as the presence of at least one allele at a given locus in a given sample.

### Population genetic analysis

An infection was defined as polyclonal if two or more alleles were observed at one or more loci. The Multiplicity of Infection (MOI) for a given sample was defined as the maximum number of alleles observed at any of the seven loci investigated. The average MOI for a study site was estimated by the mean MOI across all its samples. For all further analyses, only the predominant allele at each locus in each isolate was used [28].

Population-level genetic diversity was characterized using a measure of the expected heterozygosity (*H*_E_). *H*_E_ provides a measure of the probability that two unrelated parasites will exhibit different genotypes at a given locus. *H*_E_ was calculated for each locus using the following formula: *H*_E_ = [*n*/(*n*-1)][1-Σ*p*_*i*_^2^], where *n* is the number of isolates analysed and *p*_*i*_ is the frequency of the *ith* allele in the given population. *H*_E_ was averaged across the 7 loci in each population to provide a measure of population diversity.

The pair-wise *F*_ST_ metric was employed as a proxy of the genetic distance between pairs of populations. Calculations were undertaken using Arlequin software (version 3.5) [29]. Anticipating high marker diversity (which constrains the maximum genetic distance possible), standardized measures of the genetic distance (i.e. *F’*_ST_) were also calculated [30]. Briefly, the original *F*_ST_ value was divided by the *F*_ST-max_, the maximum possible *F*_ST_ value calculated by recoding the data such that none of the alleles were shared amongst populations. Pair-wise tests were undertaken for comparisons between Anhui and Jiangsu Province, as well as between different years.

STRUCTURE software version 2.3.3 [31] was used to determine the most likely number of populations (*K*) in the total sample, and derive the probability of ancestry of each isolate to each of the *K* populations. Model parameters were admixture with correlated allele frequencies. Twenty replicates were run for each of *K* from 1 - 10, each for 200,000 iterations (including 100,000 for burn-in). The most probable *K* was derived by calculating *ΔK* as described elsewhere [32] for each of *K* = 2-9, and using the log probability of the data [Ln P(D)]. STRUCTURE results were displayed in barplots using *Distruct* software version 1.1 [33].

After exclusion of loci with more than 15% missing data, multi-locus haplotypes (or infection haplotypes) were reconstructed from the predominant allele at each locus in isolates with no missing data at the remaining loci. Using these haplotypes, multi-locus linkage disequilibrium (LD) was measured by the standardized index of association (*I*_A_^S^) using the web-based LIAN 3.5 software [34]. Under the null hypothesis of linkage equilibrium, the significance of the *I*_A_^S^ estimates was assessed using 10,000 random permutations of the data. For each population, LD was assessed in 1) the full sample set, and 2) a curtailed sample set with each unique haplotype represented once only.

The genetic relatedness between sample pairs was assessed by a simple measure of the proportion of alleles shared between haplotype pairs (*ps*), and using (*1-ps*) as a measure of genetic distance [35]. Samples with missing data at one or more loci were excluded from analysis. An unrooted neighbour-joining tree [36] was generated from the distance matrix using the APE (Analysis of Phylogenetics and Evolution) package in R [37]. In addition, Principal component analysis (PCA) was undertaken on the distance matrix. The PCA matrix was generated using the *pca* function in the Modular toolkit for Data Processing framework in Python [38], and plotting was undertaken using matplotlib library version 1.3.1 [39].

For temporal assessment of haplotype frequency and persistence, isolates were grouped by date of collection into yearly quarters. Mantel’s r-test was used to assess the correlation between the genetic distance and temporal distance using the ade4 Package in R [40].

With the exception of the neighbour-joining tree and PCA, cases from Yunnan and Henan Provinces, and suspected internationally imported cases were not included in the analyses.

## Results

### Samples and genotyping

A summary of the number of isolates included in the study by site and year of collection is presented in Table 1. Of the 161 *P. vivax* isolates assayed, 128 (80%) could be genotyped successfully at a minimum of six of the seven loci investigated. Subsequent analysis was restricted to these 128 “pass” isolates, which included 94 isolates from Anhui, 25 from Jiangsu, two from Henan, two from Yunnan, with five internationally imported (India = 1, Nigeria = 1, Pakistan = 2, Papua New Guinea = 1).

Insufficient sample sizes were available for temporal analyses within Jiangsu Province (n < 20 in all years). However, as detailed further in the results section on “Population Structure and Differentiation”, allele sharing between Anhui and Jiangsu was sufficient to enable pooling of the two provinces for both spatial and temporal investigations in “Central China”. After pooling, sufficient sample sizes were available for comparisons between 2008, 2009 and 2010. As detailed in Table 1 and Figure 2, malaria incidence declined rapidly between 2008 and 2010 in Anhui, but remained consistently low in Jiangsu.

The genotyping assays exhibited less than 2% failure in the pass samples, apart from the MS10 and MS5 loci, which exhibited a 21% and 11% genotyping failure rate respectively. With the exception of the MS10 locus, 84% (108/128) of the pass samples exhibited a full set of genotype calls at all loci.

### Within-host diversity

Table 2 presents a summary of the percentage of polyclonal infections, multiplicity of infection (MOI) and population level diversity in Anhui and Jiangsu Provinces. When isolates from Anhui and Jiangsu were pooled (Central China), only 5.9% (7/119) of infections demonstrated evidence of multiple clones. Polyclonal infections were observed more frequently in Jiangsu (12%, 3/25) than Anhui (4.3%, 4/94) although this did not reach statistical significance at the 5% level (Fisher’s exact test, *P* = 0.160). The frequency of polyclonal infection increased over time in Central China, from 1.9% (1/54) in 2008, to 5.1% (2/39) in 2009, and 19% (4/21) in 2010 (Fisher’s exact test, *P* = 0.029).

**Table 2:** Within-host and population diversity

**Region**	**Year**	**% Polyclonal infections**	**Average MOI (range)**	**Population diversity (mean *H***_**E**_**)**
**Anhui**	2007-10	4.3% (4/94)	1.04 (1-2)	0.816
**Jiangsu**	2008-10	12% (3/25)	1.12 (1-2)	0.809
**Central China**	2008	1.9% (1/54)	1.02 (1-2)	0.816
	2009	5.1% (2/39)	1.05 (1-2)	0.796
	2010	19% (4/21)	1.19 (1-2)	0.849
**Central China**	2007-10	5.9% (7/119)	1.06 (1-2)	0.824

Measures of the MOI corresponded with the patterns observed in the frequency of polyclonal infections (Table 2). The MOI was 1.04 in Anhui, 1.12 in Jiangsu, with an overall average of 1.06 across both provinces. The maximum number of alleles observed within a sample at any of the loci investigated was 2. Only one of the seven polyclonal infections detected across Central China displayed multiple alleles at more than one marker.

### Population diversity

Population diversity was high across all isolates, heterozygosity (*H*_E_) ranging from 0.809 in Jiangsu and 0.816 in Anhui, with an overall *H*_E_ = 0.824 across both sites (Table 2). Temporally, the population diversity was moderately higher in 2010 (*H*_E_ = 0.849) compared to 2008 (*H*_E_ = 0.816) or 2009 (*H*_E_ = 0.796).

### Population structure and differentiation

As detailed in Table 3, the unadjusted *F*_ST_ between Anhui and Jiangsu was low (*F*_ST_ = 0.018). Comparably low levels of differentiation were observed between 2008, 2009 and 2010 (unadjusted *F*_ST_ range = 0.000 - 0.039). Only the differentiation between 2009 and 2010 approached significance (*F*_ST_ = 0.039, *P* = 0.018). Adjustment for the marker diversity increased the levels of differentiation between Anhui and Jiangsu and in the annual pair-wise comparisons, but the overall levels remained low to moderate (standardized *F*_ST_ range = 0.000 - 0.214).

**Table 3:** Pair-wise differentiation

**Spatial**	**Jiangsu**		
**Anhui**	0.018 (*P* = 0.081)	0.100	
**Temporal**	**2008**	**2009**	**2010**
**2008**	-	0.000	0.092
**2009**	0.000 (*P* = 0.459)	-	0.214
**2010**	0.016 (*P* = 0.117)	0.039 (*P* = 0.018)	-

Pair-wise *F*_ST_ and *F’*_ST_ values. Unadjusted *F*_ST_ values are presented in the bottom triangle, and standardized (*F’*_ST_) values are presented in the top triangle.

Using the *ΔK* method, STRUCTURE analysis suggested evidence of population sub-structure, with *K* = 4 identified as the most likely number of sub-populations (Additional file 2). However, the log probability of the data [Ln P(D)], which unlike *ΔK* includes assessment of *K* = 1, found no evidence of population substructure (Additional file 2). At *K* = 4, all four sub-populations were observed in both Anhui and Jiangsu (Additional file 3).

### Linkage disequilibrium

Moderately high linkage disequilibrium (LD) was observed in Anhui (*I*_A_^S^ = 0.261, *P* < 0.001), Jiangsu (*I*_A_^S^ = 0.404, *P* < 0.001), and across Central China as a whole (*I*_A_^S^ = 0.254, *P* < 0.001) (Table 4). Temporally, moderately high LD was observed in all years tested (*I*_A_^S^ = 0.230 - 0.322, *P* < 0.001). After accounting for outbreak samples by analysing unique haplotypes only in each test group, the *I*_A_^S^ dropped two to three-fold in each of Anhui and Jiangsu, and across Central China. In the temporal analyses, the largest reduction in *I*_A_^S^ (four-fold) was observed in 2008, with two-fold and three-fold reductions observed in 2009 and 2010, respectively.

**Table 4:** Linkage disequilibrium

		^**1**^**All samples**			**Unique haplotypes**			**Reduction in *I***_**A**_^**S**^
**Region**	**Year**	***N***	***I***_**A**_^**S**^	***P-value***	***N***	***I***_**A**_^**S**^	***P-value***	**Fold change**
**Anhui**	2007-10	84	0.261	*P* < 0.001	41	0.092	*P* < 0.001	2.8
**Jiangsu**	2008-10	17	0.404	*P* < 0.001	11	0.209	*P* < 0.001	1.9
**Central China**	2008	43	0.230	*P* < 0.001	25	0.054	*P* < 0.001	4.3
	2009	34	0.273	*P* < 0.001	20	0.151	*P* < 0.001	1.8
	2010	19	0.322	*P* < 0.001	15	0.112	*P* < 0.001	2.9
**Central China**	2007-10	101	0.254	*P* < 0.001	46	0.093	*P* < 0.001	2.7

^1^All samples with no missing data at 6 loci (excluding MS10).

### Relatedness and temporal trends

**Figure 3:**
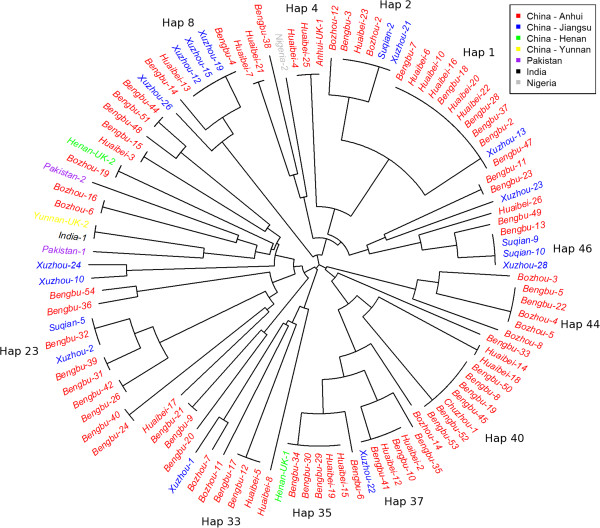
**Unrooted neighbour-joining trees illustrating genetic relatedness between isolates.** Tree generated with data from all markers with the exception of MS10. Only samples with no missing data at these loci are presented, including 103 central Chinese, 1 southern Chinese, and 4 imported isolates. Colour-coding reflects the province (first administrative level) for the Chinese isolates, and country for the imported isolates. Further geographic information on the origin of the Chinese isolates is provided in the sample labels which refer to the prefecture (second administrative level). Multi-locus haplotypes observed 3 or more times are labelled.

A neighbour-joining tree based on a pair-wise distance matrix at all loci with the exception of MS10 was generated using the data from 104 Chinese samples (Anhui = 84, Henan = 2, Jiangsu = 17, Yunnan = 1) and four suspected imported cases (India = 1, Nigeria = 1, Pakistan = 2) with no missing data (Figure 3). A total of 50 different 6-locus haplotypes were observed in the data set. Several clusters of samples with identical haplotypes were observed in both Anhui and Jiangsu. The largest cluster of identical samples (Haplotype 1) comprised twelve isolates sourced from two prefectures in Anhui and one in Jiangsu. Ten other haplotypes (Haplotypes 2, 4, 8, 23, 33, 35, 37, 40, 44 and 46) were observed three or more times (range 3 - 7) in the data set. Each of these clusters again, generally represented a range of prefectures.

**Figure 4:**
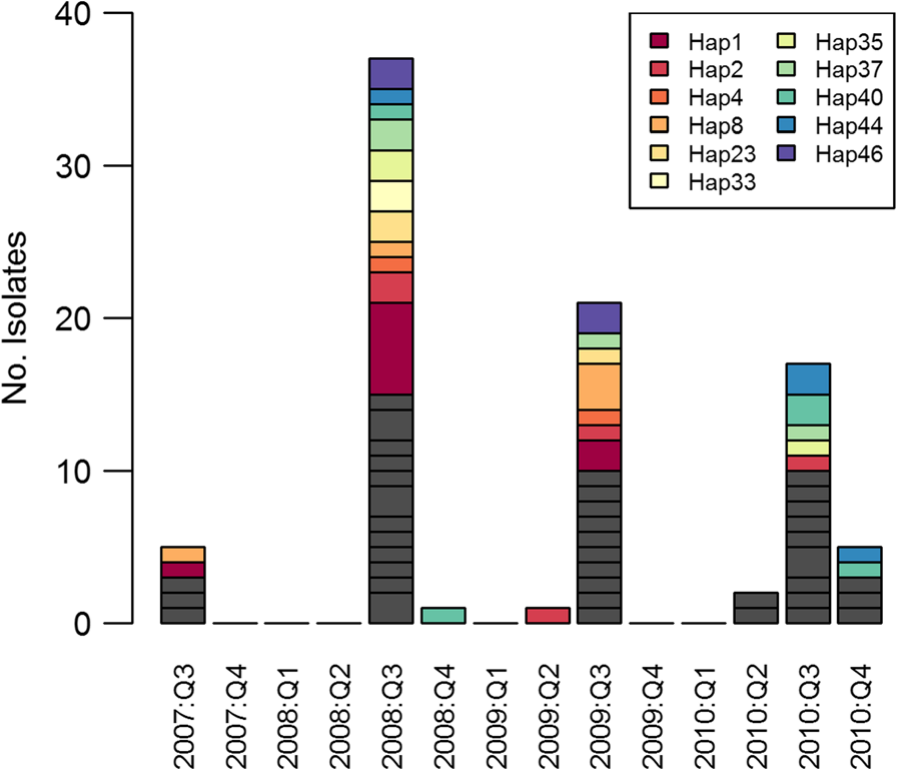
**Temporal haplotype dynamics.** Frequencies of multi-locus haplotypes reconstructed from the genotypes at all loci with the exception of MS10 are presented by quarter. For simple visual representation, only haplotypes observed 3 or more times in the full data set are presented in colour, whilst all other (low frequency) haplotypes are presented in gray. Note - exact dates of collection (only year of collection) were not available for nineteen of the samples from Anhui with full data at the loci investigated. These isolates (including three from 2009 with Hap1, one 2009 with Hap4, one 2008 with Hap33, three 2009 with Hap35, and two 2009 with Hap40) were therefore not presented in the plot.

A weak but significant correlation was found between genetic distance (estimated from the number of genotype differences) and temporal distance classified by annual quarter (Mantel r-test, r = 0.05, *P* = 0.01). Further visual representation of haplotype dynamics over time is presented in Figure 4. The majority of the haplotypes observed three or more times (10/11) were observed in two or three different years.

### Relatedness of imported cases

Visual inspection of the neighbour-joining tree (Figure 3) and principal components analysis (PCA) plot (Additional file 4) did not reveal any distinct clustering of the isolates from Yunnan Province, Southern China, or the imported cases (India, Pakistan and Nigeria) from the central Chinese isolates. The Indian isolate shared an identical multi-locus haplotype with one of the two isolates from Yunnan. The next most closely related isolate to this pair of infections was one of the two imported cases from Pakistan. The imported cases from South Africa and Papua New Guinea could not be plotted on the neighbour-joining tree or PCA plot owing to incomplete genotype profiles.

## Discussion

In Central China, the epidemiology of *P. vivax* malaria has changed significantly over the past couple of decades, with overall declining incidence interrupted by epidemic expansions and changing dynamics of imported cases. These epidemiological changes may impact the transmission dynamics of the local *P. vivax* population. Indeed, we recently demonstrated unstable, epidemic *P. vivax* transmission in the pre-elimination setting of Sabah, Malaysia, which we hypothesized to be largely affected by the changing epidemiological dynamics in the rapidly shrinking parasite population [10]. Previous studies have used genotyping of variable surface antigens to describe the diversity of *P. vivax* populations in Central China in the mid to late 2000s for the purposes of assessing a potential vaccine candidate [41-43]. However, assessments of *P. vivax* population structure and transmission dynamics, requiring neutral genetic markers, have not yet been described in this region. The current analysis focused on the population structure and transmission dynamics of *P. vivax* isolates in Central China using neutral genetic markers on parasites collected over the past few years following the most recent resurgence, presenting evidence of moderately unstable, largely clonal transmission in a parasite population with high levels of diversity. This data set should provide a useful baseline against which to compare patterns of *P. vivax* diversity and structure in Central China in later years in order to assess the impact of ongoing transmission intervention efforts in the region, as recently demonstrated in Sri Lanka [44].

Parasite isolates were sourced from two neighbouring provinces in Central China (Anhui and Jiangsu). Anhui exhibits the highest incidence of malaria in Central China (API 5.0 - 0.28 between 2007 and 2010), significantly greater than Jiangsu (API 0.05 - 0.09 between 2007 and 2010). The latter has not experienced any indigenous malaria cases in 2012 and 2013. Only moderate genetic differentiation was observed between the two provinces and this was apparent even after adjusting for the extensive marker diversity (*F’*_*ST*_ = 0.1). However, the number of isolates from Jiangsu was small, reducing the power of the analysis. Nonetheless, in accordance with the *F’*_*ST*_ results, limited evidence for differentiation between Anhui and Jiangsu was observed with STRUCTURE analysis, with any sub-populations rather being shared amongst the two provinces. The level of differentiation observed with the *F’*_*ST*_ analysis was lower than levels observed in intra-country comparisons in Sabah (*F’*_*ST*_ = 0.5 - 0.6) [10] and Colombia (*F’*_*ST*_ = 0.4 - 0.7) [45], where *P. vivax* endemicity is comparably low. Rather, the differentiation is more comparable to levels observed between sites in Papua New Guinea (PNG) (*F’*_*ST*_ = 0.14 - 0.16) [46]. The mechanisms responsible for these observations in Central China are likely to be different from those in PNG, where transmission intensity is considerably higher, relapse dynamics differ, and human movement between populations may be limited. In contrast to the marked geographical boundaries in PNG, the majority of the Chinese isolates were sourced from neighbouring prefectures with limited geographical boundaries. This is not unexpected as hundreds and thousands of people travel between the two provinces every day. The recent epidemic expansion in Central China may have added to the increased panmixis between the provinces affected. In addition, the long dormancy duration (often exceeding eight months) of the temperate *P. vivax* strains endemic to Central China may have contributed to the persistence and spread of infections both temporally and geographically. Indeed, the majority of the multi-locus haplotypes observed three or more times in the data set (10/11), were observed in two or three different years of collection. Hence the current evidence suggests that the recently malaria-free province of Jiangsu is at moderately high risk of re-introduced malaria from Anhui.

The current analysis of seven STR markers demonstrated polyclonal infections to be infrequent in both Anhui (4%) and Jiangsu (12%). These levels of polyclonal infection are generally lower than those observed in tropical and sub-tropical endemic populations across the globe [24,26,27,45-49], possibly reflecting the different relapse dynamics and relatively short window for local transmission in the more temperate setting of Central China. However, caution is required in comparison between different study sites owing to differences in markers and/or potential differences in allele calling. Previous analysis of *P. vivax* surface antigens on isolates from Anhui Province demonstrated a low frequency of polyclonal infections in 2004 (6%) [42], and moderate frequency (15%) between 2006 and 2008 [43]. Across both Anhui and Jiangsu, a trend of increasing frequency of polyclonal infections was observed between 2008 and 2010 (2 - 19%). The results across the studies agree on a generally low frequency of polyclonal infections in Central China, compatible with largely clonal transmission dynamics over the past 10 years. However, the factor(s) responsible for the subtle variation between Anhui and Jiangsu, and temporally, remain unclear. It might be speculated that infections introduced from regions with more “tropical” relapse dynamics are responsible for some of the variation. Indeed, the high relatedness (maximum of one multi-allelic locus) between the clones in the polyclonal infections detected may support a relapse versus super-infection dynamic. Clonal transmission dynamics during expansions may have also contributed to the observed trends. However, firm conclusions can not be made owing to the limited sample size. Further investigations with larger sample size are required to confirm the trend and to elucidate the relative impact of relapse and imported/introduced cases on polyclonal infection dynamics in Central China.

The results of the analyses of population diversity and linkage disequilibrium (LD) demonstrate evidence of moderate instability in *P. vivax* transmission in Central China. As observed in *P. vivax* populations from multiple endemic settings across the globe [24,26,27,45-49], high levels of diversity were observed in both Anhui and Jiangsu (*H*_E_ = 0.8). The reservoirs sustaining this diversity in the face of aggressive containment efforts remain unclear. Sub-patent and asymptomatic infections may present a largely hidden reservoir, with undetected imported infections in particular enabling maintained diversity. Indeed, the propensity of *P. vivax* to relapse from its dormant liver stage weeks to months after the primary infection may greatly aid the introduction of imported strains. A recent study investigating temporal trends in the diversity of *P. vivax* isolates in Sri Lanka demonstrated that despite rapidly declining endemicity between 2003-2004 and 2006-2007, population diversity remained unexpectedly high in the latter years [44]. The authors postulated that imported infections were likely to be a major source of the maintained population diversity in this setting. In tropical and subtropical regions with year round transmission, outcrossing may enhance the population diversity. Indeed, in the low endemic setting of Temotu Province, Solomon Islands, where a recent study demonstrated comparatively higher diversity in *P. vivax* (*H*_E_ = 0.85) relative to *P. falciparum* (*H*_E_ = 0.54), frequent relapse may have facilitated polyclonal infection and outcrossing in the *P. vivax* population, with moderate contribution to the observed diversity [26]. However, as demonstrated in South Korea [50], the narrow window for transmission in temperate regions reduces the opportunity for recombination and, thus, is not likely to be a major source maintaining the diversity in Central China.

Despite the high population diversity, LD remained high in both Anhui and Jiangsu and in each year tested (*I*_A_^S^ = 0.2 – 0.4), suggestive of low recombination, as might be anticipated with largely clonal transmission and limited opportunity for transmission. However, when each distinct haplotype was represented just once, the strength of LD (*I*_A_^S^) declined two- to four-fold, with the greatest decline observed in 2008 (shortly after the peak of the *P. vivax* resurgence in Central China). The strengthening of LD by the expansion of a few haplotypes in an otherwise moderately panmictic population is suggestive of occasional outbreaks/epidemic transmission. Indeed, multiple small outbreak clusters of identical haplotypes were observed in the neighbour-joining plot. In Sabah, similar outbreak dynamics were observed, but with the majority of the identical haplotypes observed within short temporal windows and moderately confined geographic space [10]. In contrast, identical Chinese haplotypes were often observed across multiple years (Figure 4) and prefectures (Figure 3). Indeed, only a weak correlation was observed between the genetic and temporal distance between the Chinese isolates (Mantel r-test, r = 0.05). Variation in relapse dynamics between the tropical Sabahan isolates, and the temperate Chinese isolates may account in part for these differences.

In settings such as Central China, where the endemicity of local infections is declining rapidly but the relative proportion of imported cases rising, geographic markers enabling confirmation of imported versus local *P. vivax* cases are urgently needed. Using a selection of samples sourced from *P. vivax* cases acquired from Southern China and internationally, a provisional assessment was undertaken on the utility of the current marker panel to distinguish central Chinese from imported infections. As demonstrated with neighbour-joining analysis and principal component analysis, although none of the isolates from outside Central China shared identical haplotypes to any of the central isolates, evidence for the utility of the marker panel for detecting imported cases was limited. A recently developed data analysis platform (VivaxGEN) has been made available for enhanced comparison of STR-based *P. vivax* data between studies [51]. This multi-centre approach enables data sharing between Central China and other *P. vivax* endemic regions across the globe, so that the utility of the APMEN marker panel as well as new markers can be investigated comprehensively. Indeed, as recently demonstrated in a global study of variation in the *P. vivax* mitochondrial genome, SNPs in this organelle may offer some assistance in resolving the geographic origin of isolates at least at a regional level [52]. Furthermore, variants in the apicoplast genome may enable further geographic resolution as recently demonstrated in a global selection of *P. falciparum* isolates [53].

## Conclusions

Although interventions to suppress the recent *P. vivax* resurgence in Central China appear to have been successful in reducing the parasite incidence, genotyping analysis confirms complex parasite dynamics during the suppression phase. The ability of certain parasite lineages to expand locally demonstrates the instability of transmission and risk of future resurgence. With limited barriers to gene flow between Anhui and Jiangsu, the risks of resurgence in the latter demand continued dedication to surveillance to detect early warning signals. Parasite genotyping may facilitate early detection of outbreaks. Further studies utilising the consensus marker sets or barcodes informed by nuclear and organellar genomic data will be required to discriminate local from imported *P. vivax* isolates in Central China and other vivax endemic regions. Such studies will inform the main reservoirs of infection, the degree to which imported cases may affect the local transmission, and in doing so help to focus appropriate malaria control resources to ensure the ultimate elimination of the parasite.

## Competing interests

The authors declare that they have no competing interests.

## Authors’ contributions

YL, QG, QC, RH, SA, JC and RNP conceived and designed the study. YL, JC, and KG performed the laboratory assays. HT, SA, TGC, YL and JC performed the data analysis. JC, YL, QG and HYZ provided essential contribution to the sample collections. YL, SA, QC, RNP, HT, JC, RH and QG wrote the manuscript and facilitated data interpretation. All authors read and approved the final manuscript.

## Supplementary Material

Additional file 1 Assay details. Click here for file

Additional file 2 **Inference of most likely STRUCTURE-defined *K *** **in Anhui and Jiangsu.** The top panel presents *ΔK* against *K* (from 2-9: cannot evaluate the first and last *K*), with weak evidence for the most likely *K* of 4 [32]. The bottom panel presents the mean log probability of the data against *K* (from 1 - 10), with weak evidence for the most likely *K* at 1. Click here for file

Additional file 3 **Population structure in Anhui and Jiangsu inferred by STRUCTURE at *K* = 4. **Each vertical bar represents a sample, with colour-coding reflecting the predicted ancestry to each of the 4 (K) sub-populations. K1 = light green, K2 = dark green, K3 = red, and K4 = orange. Click here for file

Additional file 4 **Principal component analysis of the genetic variation between local and imported *P. vivax* cases in Central China.** The PCA was generated using data from 104 Chinese and 4 imported isolates which exhibited full genotype profiles at the loci investigated (excluding MS10). As with the neighbour-joining tree (Figure 4), colour-coding reflects the province (first administrative level) for the Chinese isolates, and country for the imported isolates. Click here for file
