# Supplementary material for: Genetic diversity and population structure of Plasmodium vivax in Central China
Source: Malar J. 2014 Jul 9;13:262. doi: 10.1186/1475-2875-13-262 (PMC4094906; doi:10.1186/1475-2875-13-262)
Supplement: Additional file 1 — Assay details. [file 1475-2875-13-262-S1.doc]

| Marker | Primer | Sequence (5'-3') |
| --- | --- | --- |
| MS1 | Forward | **6FAM**-TCAACTGTTGGAAGGGCAAT |
|  | Reverse | ctgtcttTTGCTGCGTTTTTGTTTCTG |
| MS10 | Forward | **PET**-TTATCCCTGCTGGATGTGAA |
|  | Reverse | ctgtcttTCCTTCAGGTGGGACTTGTT |
| MS5 | Forward | **NED**-CGTCCTCTATCGCGTACACA |
|  | Reverse | ctgtcttAAAGGGAGAGGAGCGAAAAC |
| MS8 | Forward | **VIC**-AGAGGAGGCAGAAATGCAGA |
|  | Reverse | ctgtcttAGCCCCTTTGCGTTCTTTAT |
| Pv3.27 | Forward Primary | TTTTTCAACTTGCTGCCCCCTG |
|  | Forward Nest | **6FAM**-GGACATTCCAAATGTATGTGCAGTCG |
|  | Reverse | *CGTCATCGTCATTGCTCTGGAG |
| MS16 | Forward Primary | TTCCTGATGACAATTTCGACGG |
|  | Reverse Primary | TCTCTTCCCATTTGAGCATCGC |
|  | Forward Nest | **PET**-CTTGTTGTGGTTGTTGATGGTG |
|  | Reverse Nest | *AGTACGTCAACCATGTGGGTAG |
| msp1F3 | Forward Primary | GGAGAACATAAGCTACCTGTCC |
|  | Reverse Primary | GTTGTTACTTGGTCTTCCTCCC |
|  | Forward Nest | **VIC**-CAAGCCTACCAAGAATTGATCCCCAA |
|  | Reverse Nest | *ATTACTTTGTCGTAGTCCTCGGCGTAGTCC |

*5’ 7bp adenine tail (‘ABDTAIL’)
