# Supplementary figures and images for: Genetic diversity and population structure of Plasmodium vivax in Central China
Source: Malar J. 2014 Jul 9;13:262. doi: 10.1186/1475-2875-13-262 (PMC4094906; doi:10.1186/1475-2875-13-262)

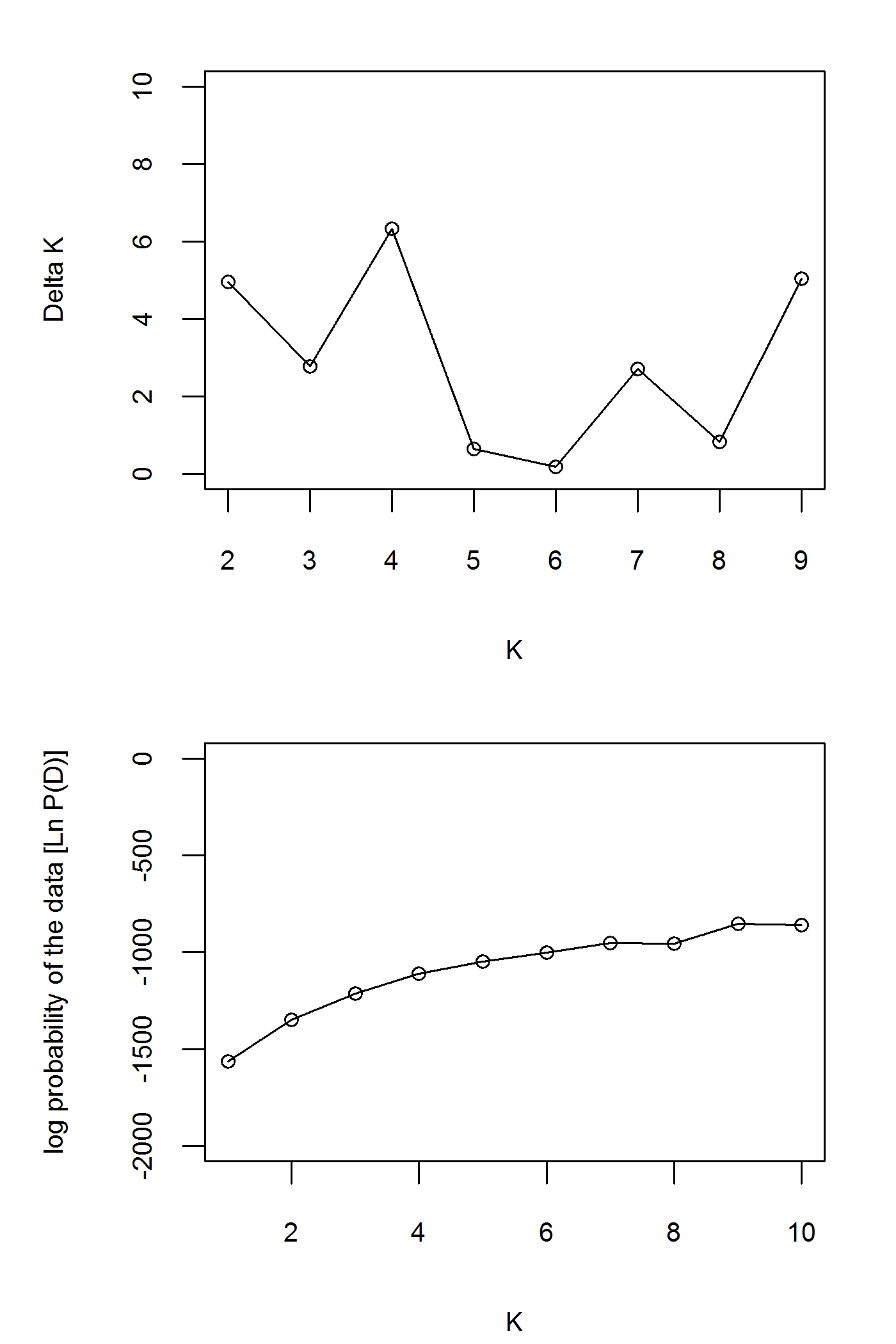

Supplement: Additional file 2 — Inference of most likely STRUCTURE-defined K in Anhui and Jiangsu. The top panel presents ΔK against K (from 2-9: cannot evaluate the first and last K), with weak evidence for the most likely K of 4 [32]. The bottom panel presents the mean log probability of the data against K (from 1 - 10), with weak evidence for the most likely K at 1. [file 1475-2875-13-262-S2.tiff]

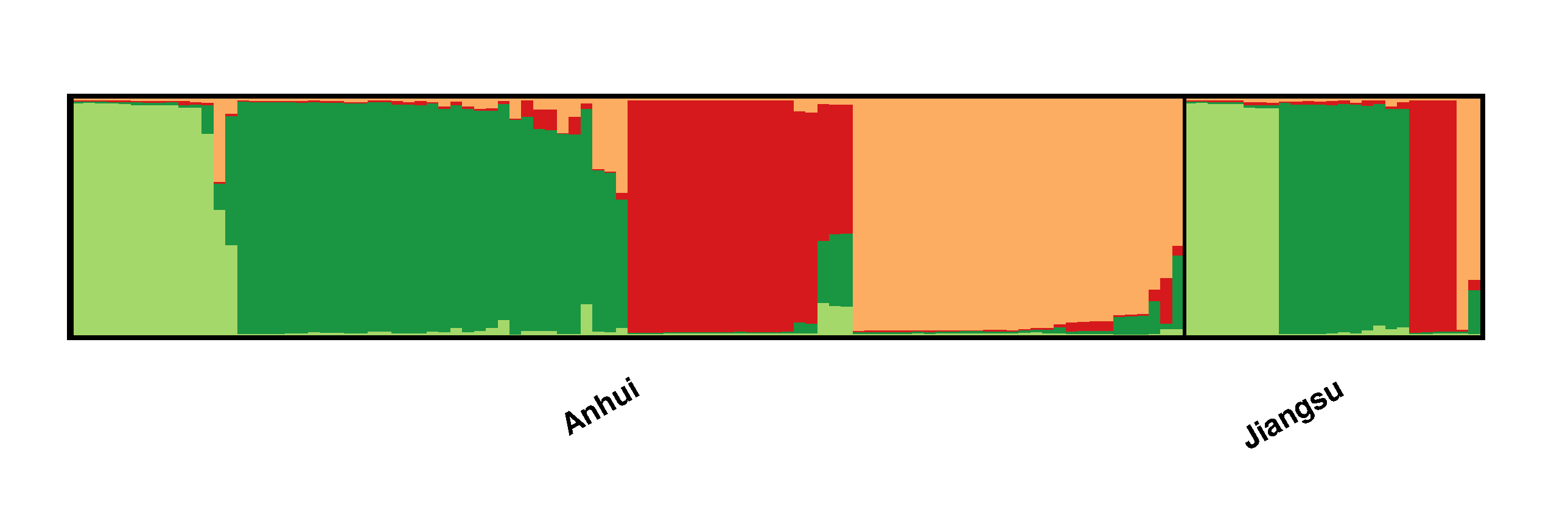

Supplement: Additional file 3 — Population structure in Anhui and Jiangsu inferred by STRUCTURE at K = 4. Each vertical bar represents a sample, with colour-coding reflecting the predicted ancestry to each of the 4 (K) sub-populations. K1 = light green, K2 = dark green, K3 = red, and K4 = orange. [file 1475-2875-13-262-S3.tiff]

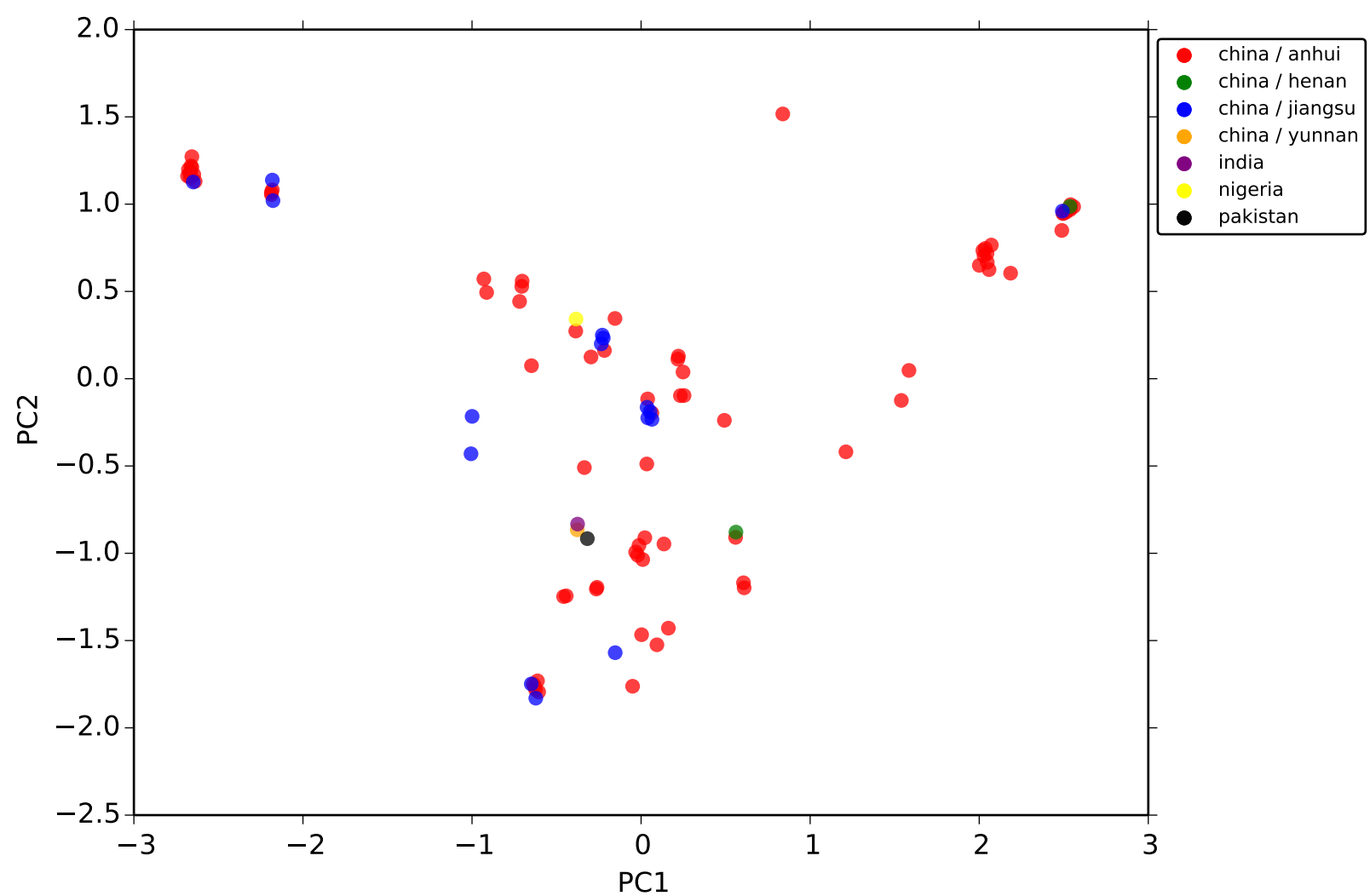

Supplement: Additional file 4 — Principal component analysis of the genetic variation between local and imported P. vivax cases in Central China. The PCA was generated using data from 104 Chinese and 4 imported isolates which exhibited full genotype profiles at the loci investigated (excluding MS10). As with the neighbour-joining tree (Figure 4), colour-coding reflects the province (first administrative level) for the Chinese isolates, and country for the imported isolates. [file 1475-2875-13-262-S4.pdf]
